# Circadian profiling in two mouse models of lysosomal storage disorders; Niemann Pick type-C and Sandhoff disease

**DOI:** 10.1016/j.bbr.2015.10.021

**Published:** 2016-01-15

**Authors:** Katie Richardson, Achilleas Livieratos, Richard Dumbill, Steven Hughes, Gauri Ang, David A. Smith, Lauren Morris, Laurence A. Brown, Stuart N. Peirson, Frances M. Platt, Kay E. Davies, Peter L. Oliver

**Affiliations:** a Medical Research Council Functional Genomics Unit, Department of Physiology, Anatomy and Genetics, University of Oxford, Parks Road, Oxford, OX1 3PT, UK; b Nuffield Department of Ophthalmology, Nuffield Department of Clinical Neurosciences, University of Oxford, West Wing, John Radcliffe Hospital, UK; c Department of Pharmacology, University of Oxford, Mansfield Road, Oxford, UK

**Keywords:** Lysosome storage disorder, Ataxia, Mouse mutant, Circadian

## Abstract

Sleep and circadian rhythm disruption is frequently associated with neurodegenerative disease, yet it is unclear how the specific pathology in these disorders leads to abnormal rest/activity profiles. To investigate whether the pathological features of lysosomal storage disorders (LSDs) influence the core molecular clock or the circadian behavioural abnormalities reported in some patients, we examined mouse models of Niemann-Pick Type-C (*Npc1* mutant, *Npc1^nih^*) and Sandhoff (*Hexb* knockout, *Hexb^−/−^*) disease using wheel-running activity measurement, neuropathology and clock gene expression analysis. Both mutants exhibited regular, entrained rest/activity patterns under light:dark (LD) conditions despite the onset of their respective neurodegenerative phenotypes. A slightly shortened free-running period and changes in *Per1* gene expression were observed in *Hexb^−/−^* mice under constant dark conditions (DD); however, no overt neuropathology was detected in the suprachiasmatic nucleus (SCN). Conversely, despite extensive cholesterol accumulation in the SCN of *Npc1^nih^* mutants, no circadian disruption was observed under constant conditions. Our results indicate the accumulation of specific metabolites in LSDs may differentially contribute to circadian deregulation at the molecular and behavioural level.

## Highlights

• The circadian behaviour of two models of lysosomal storage disorders were examined.
• *Npc1* mutant mice show no core circadian defects.
• *Hexb* knockouts show potential circadian activity disturbances.
• Contrasting pathology in LSDs may differentially contribute to circadian regulation.

## 1 Introduction

Lysosomal storage disorders (LSDs) constitute a large group of inherited metabolic diseases that typically involve pathology in the central nervous system [1], [2]. Defects in acidic hydrolases, lysosomal membrane proteins or non-enzymatic soluble lysosomal proteins underlie the majority of LSDs, often resulting in the accumulation of incompletely metabolised macromolecules inside organelles of the endosomal–autophagic–lysosomal system [3], [4]. For example, mutations in genes encoding the proteins deficient in Niemann-Pick disease, type-C1 (*NPC1)* or type-C2 *(NPC2)* lead to severe neurodegeneration and liver dysfunction [5], [6], [7]. In addition, Sandhoff disease is a related LSD that arises from mutations in the β-subunit of the lysosomal enzyme β-hexosaminidase (*HEXB*) with a resultant accumulation of GM2 gangliosides in the endolysosomal compartment and severe neuronal degeneration [2]. Both these disorders are typical amongst LSDs in that the neurological symptoms are a major feature of the disease [8], including ataxia, motor deterioration and cognitive defects [2].

Circadian rhythm disruption is frequently observed in neurological disorders and it is becoming apparent that sleep disturbance is a potentially important prodromal symptom in neurodegenerative disease [9], [10], [11]; for example, rapid eye movement sleep behaviour disorder (RBD) is present years before the diagnosis of Parkinson's disease in up to 50% of patients [12], [13]. Similar observations have been documented in LSDs, including patients with juvenile neuronal ceroid lipofuscinosis and Sanfilippo syndrome, where sleep disruption including irregular sleep/wake patterns are commonly reported [14], [15], [16]. With particular relevance this study, sleep disruption has been described as a characteristic of patients with infantile and juvenile GM2 gangliosidosis [17], [18]. Furthermore, in Niemann-Pick disease, cataplectic attacks have been associated with excessive daytime sleepiness [19], in addition to altered sleep efficiency and reduced sleep time – including REM and delta sleep – compared to age-matched controls [20]. A more recent study has identified restless and disturbed sleep in both infantile and adult onset NPC1 patients; however, sleep problems are often over-looked and under-reported which in part may be attributed to the severe course of the disease [21]. Indeed, emerging evidence highlights the link between the circadian clock and metabolic pathways [22], [23]; however, it is unclear whether the cellular defects associated with metabolic disorders such as LSDs influence directly the core molecular clock and thus sleep/wake activity and timing.

In order to investigate whether the specific pathologies related to the LSDs NPC and Sandhoff disease influence circadian behaviour, we examined mouse models of both these disorders, the *Npc1* null mutant (*Npc1^nih^*) and *Hexb* knockout (*Hexb^−/−^*) [24], [25]. For the first time, rest/activity profiles were assessed in these mutants using wheel-running activity measurement in addition to neuropathological and clock gene expression analysis. Interestingly, our data suggests that individual cellular pathways affected in LSDs may contribute to some aspects of circadian deregulation at the behavioural and molecular level.

## 2 Materials and methods

### 2.1 Mice

*Npc1^nih^* (BALB/cNctr-*Npc1^m1N^*) mutant mice contain a retroposon insertion leading to a complete absence of the protein in homozygous animals [25]. Mutants show some subtle motor-coordination defects at 7–8 weeks of age, followed by progressive ataxia from 9 weeks, before reaching disease end-stage at 10–12 weeks [26], [27]. *Hexb^−/−^* mutant mice were originally generated by introduction of a neomycin-resistant vector disrupting exon 2 of the gene [24]; these mice show impaired motor-coordination from 10 weeks of age, followed by progressive ataxia and disease end-stage at 15–16 weeks [28]. All mice were maintained by heterozygous mating. Animal procedures were conducted using protocols approved by the UK Animals (Scientific Procedures) Act (1986).

### 2.2 Circadian wheel-running screens

Age and sex-matched (7-week old) *Npc1^nih^* and *Hexb^−/−^* animals (*n *= 5–7 per genotype per behavioural paradigm) with wild-type littermate controls (WT) were housed individually in cages fitted with running wheels (Coulbourn Instruments) maintained at constant temperature and humidity, with *ad libitum* food and water. Up to 6 cages were arranged in light-controlled chambers with externally controlled white LED lighting set at 150 lux at the cage floor level. The behavioural screens that were applied to examine common circadian paradigms are shown in Fig. S1. In the first *Npc1^nih^* screen (screen 1), animals were entrained to a 12:12 h light–dark (LD) cycle for 12 days (zeitgeiber time (lights on) (ZT)0 = 0600) followed by one-hour light pulse at ZT14. The mice were then placed in constant darkness (DD) for a further 10 days or until mutants reached disease end-stage. In the second *Npc1^nih^* screen with an independent cohort of animals (screen 2), mice were entrained to a 12:12 h LD cycle for 12 days (ZT0 = 0600) followed by being subjected to a six-h phase advance (ZT0 = 0000) and allowed to re-entrain under these conditions up to 10 days. In the *Hexb^−/−^* screen, mutant and WT littermate mice were entrained to a 12:12 h LD cycle for 12 days (ZT0 = 0600) followed by one-h light pulse at ZT14. The mice were then placed in DD for a further 11 days followed by constant light (LL; 150 lux) for 11 days. Data were analysed using Actimetrics Clocklab toolbox for Matlab prior to additional statistical analysis. The circadian period (tau) in LD and DD was quantified by fitting one regression line through 7 consecutive activity onsets. LD data from both *Npc1^nih^* screens was pooled prior to analysis.

### 2.3 Passive-infrared (PIR) sensor screening

PIR sensor-based actigraphy was carried out essentially as previously described [29], with full details expected to be published in the near future (Brown LA et al. in preparation). Briefly, mice were singly housed in the same conditions as described above in cages 44 cm long × 15 cm wide × 12 cm high with an acrylic block to prevent undetected movement under the food hopper. Raw data from the PIR motion sensors took the form of percentage time active per 10 second epoch, with sensors activated both by gross locomotion and small movements such as turning of the body. A cohort of age- and sex-matched (8.5-week old) *Npc1^nih^* and WT littermate controls (*n *= 5 per genotype) were monitored under a 12:12 LD cycle with white LED illumination at 150 lux.

### 2.4 Quantitative PCR (RT-qPCR)

Age-matched (7-week old) *Hexb^−/−^* (*n *= 5) and WT littermate controls (*n *= 5) were individually housed and entrained to a 12:12 LD cycle at 150 lux for 14 days prior to the day of tissue collection at ZT6. An independent cohort of *Hexb^−/−^* mutants (*n *= 4) and WT littermate controls (*n *= 5) were dark-adapted for 48 h prior to tissue collection to examine the endogenous core clock. Following cervical dislocation, livers were dissected and frozen on dry ice. Brains were immediately sectioned on a steel matrix between bregma 0 and 1 mm posterior. Tissue punches were taken using a 1 mm sample corer (FST Ltd.) from the SCN and then snap-frozen. Total RNA was extracted from the SCN and liver tissue using the RNeasyMicro or RNeasyMini kit (Qiagen), respectively. RNA quality was assessed on a 2100 BioAnalyzer using the RNA 6000 Pico Assay (Agilent Technologies) and all samples had RNA integrity (RIN) values over 7.5. SYBR green master mix (Invitrogen) RT-qPCR reactions were run in triplicate on a StepOne Real Time PCR System (Applied Biosystems), with gene expression values calculated using the 2^−ΔΔ^Ct calculation and normalised to *Gapdh* expression. Primer sequences and further details of the qPCR products are shown in Table S2.

### 2.5 Brain pathology

Lipid accumulation in *Hexb^−/−^* mice was examined in 15 μM frozen tissue sections stained for Periodic Acid Schiff (PAS), according to the manufacturer's instructions (Sigma). Filipin staining was carried out using a 0.05 mg/ml solution (Sigma) on frozen sections for 2 h at room temperature and visualised by fluorescent microscopy.

### 2.6 Retinal histology

Following enucleation whole eyes from *Hexb^−/−^* mice (*n *= 3) and WT littermate controls (*n *= 3) were snap frozen on dry ice and stored at −80 °C. Eyes were then post fixed in 4% methanol free paraformaldehyde in PBS for 16 h. Subsequent immunostaining of retina cryostat sections and whole retina flatmounts was performed as described previously [30], [31]. Rabbit polyclonal anti-melanopsin antibody (1:2500, UF006, advanced Targeting Systems) and goat polyclonal anti-Brn3a antibody (1:1000, sc-31985, Santa Cruz Biotech) were incubated overnight (retinal sections) or for 3 days (retina flatmounts) at 4 °C in PBS with 0.2% Triton-X and 2% donkey serum. Donkey anti-rabbit Alexa 568 and donkey anti-goat Alexa 488 secondary antibodies diluted in PBS 0.2% Triton-X and 2% donkey serum (1:200) were incubated for 2 h at 22 °C. For staining of retina flatmounts levels of Triton-X were increased to 1%. All wash steps were performed using PBS with 0.05% Tween-20. Samples were mounted in Prolong Gold anti-fade mounting media containing DAPI (Life Technologies).

### 2.7 Image acquisition

To examine retinal histology fluorescent images were collected using a LSM 710 laser scanning confocal microscope and Zen 2009 image acquisition software (Zeiss). Individual channels were collected sequentially. Laser lines for excitation were 405 nm, 488 nm and 561 nm. Emissions were collected between 440–480, 505–550, and 580–630 nm for blue, green and red fluorescence respectively. For all images, global enhancement of brightness and contrast was performed using Zen Lite 2011 image analysis software (Zeiss). Images of retina flatmounts represent maximum intensity projections generated from confocal slices images collected every 2.5 μm, spanning from the ganglion cell layer to the inner plexiform layer. For direct quantitative comparisons all images were acquired and processed under identical conditions.

### 2.8 Statistics

Statistics for circadian wheel running, RT-qPCR analysis, and immunofluorescence quantification were carried out using GraphPad Prism version 5.0d (GraphPad Software) or SPSS (version 20, IBM). Results are presented as mean ± SEM. All pairwise comparisons were analysed with Bonferroni post-hoc analysis. The effects of genotype and multivariate experiments were analysed using ANOVA with Bonferroni post-hoc analysis. Differences were considered to be statistically significant at *p*-values <0.05.

## 3 Results

### 3.1 Circadian behavioural screening

Initially we used home-cage wheel-running to determine the effect of loss-of-function *Npc1* and *Hexb* mutations on several aspects of circadian behaviour by comparing *Npc1^nih^* and *Hexb^−/−^* mutant mice with their respective WT littermate controls. Due to the rapid, progressive nature of neuropathology in both these models, the wheel-running screens were initiated at 7 weeks of age to limit the influence of the known gait abnormalities on data acquisition [28], [32]. In addition, the lengths of the circadian behavioural paradigms themselves were adapted to account for the limited lifespan of these mutants; not all experimental conditions could be tested in an individual mutant. As such, subsequent experimental conditions were chosen based on their phenotype in the initial screen. For example, two independent cohorts of *Npc1^nih^* mice were required to investigate both entrainment and re-entrainment (see Materials and Methods and Fig. S1 for details of the screens).

**Fig. 1:**
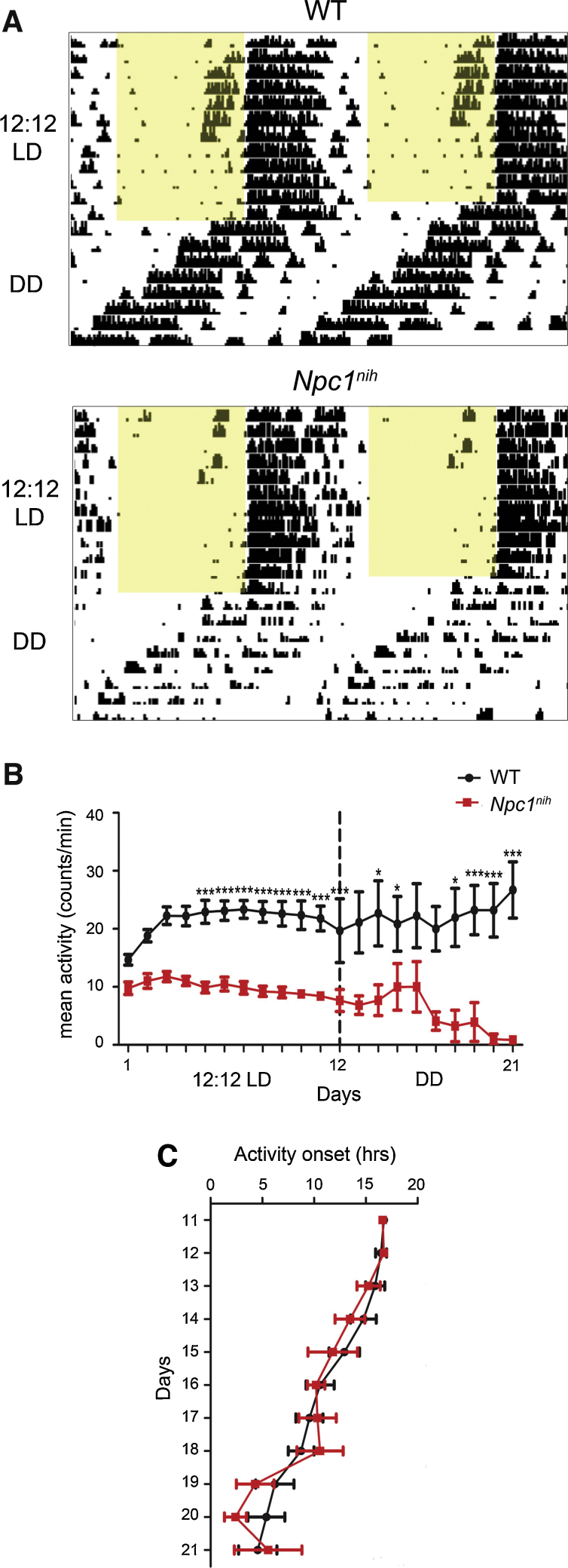
Wheel-running behaviour in *Npc1^n^^ih^* mutants in LD and DD. (A) *Npc1^nih^* activity under a 12:12 LD cycle (from 7 weeks of age) followed by DD (*Npc1^nih^* screen 1; Fig. S1 for details) versus WT controls. Each horizontal line represents a double-plot of the activity profile recorded over 24 h. Activity bars are displayed in 10 min bins and yellow shading indicates light exposure. (B) *Npc1^nih^* mutants show significantly reduced activity over each day in LD (*n* = 11 each genotype, *Npc1^nih^* screens 1 and 2 combined) and DD (*n* = 5, *Npc1^nih^* screen 1) compared to WT controls. (C) Free-running activity onsets do not differ between *Npc1^nih^* mutants and WT controls (*n* = 5, *Npc1^nih^* screen 1). Data presented as mean ± SEM; *p < 0.01, ***p < 0.0001, ANOVA.

Initially we monitored *Npc1^nih^* mice for their the entrainment to a 12:12 light/dark (LD) cycle (zeitgeiber time (ZT)0 = 0600 hr) (Fig. 1A). *Npc1^nih^* mice were able to run on the wheels and could entrain to this regular 24-h lighting schedule, with no significant difference in the time of activity onset, circadian period, or proportion of activity in the light phase compared to their respective WT controls (Fig. 1A–C and Table 1). However, the length of the active phase (alpha) for mutants was significantly shorter compared to WT mice even during the first few days of activity measurements (Fig. 1A and Fig. S2A); this demonstrates that wheel-running is able to quantify behavioural alterations prior to the onset of visible ataxia in this model [26]. In addition to the LD entrainment data, we examined negative masking using a one-h light pulse administered during LD at ZT14 (Fig. S1). All mice tested demonstrated normal negative masking responses to light as shown by suppression of their activity, suggesting light input pathways were functioning normally at 8–9 weeks of age in *Npc1^nih^* mice (Fig. S3). We next investigated free-running activity patterns under constant conditions. Under constant dark (DD), the free-running period (tau) of *Npc1^nih^* mice could be clearly defined from the onset of activity (Fig. 1A); however, this was not significantly different from WT controls (Fig. 1C); these data suggest the core clock is functioning normally in mutant animals. Further examination of wheel-running in *Npc1^nih^* animals over all phases of the circadian screen demonstrated fragmented activity compared to WT mice, as demonstrated by a small increase in the number of activity bouts per day (Fig. S2B). This was accompanied by hypoactivity in *Npc1^nih^* mice, as predicted by their progressive ataxia, with mutants showing a significant reduction in wheel-running under LD and DD; this effect became more pronounced as the mice reached disease end-stage at 10–11 weeks of age (Fig. 1B). Together, these data suggest that *Npc1^nih^* mutants show reduced wheel-running capability but have no overt circadian behavioural abnormalities.

**Fig. 2:**
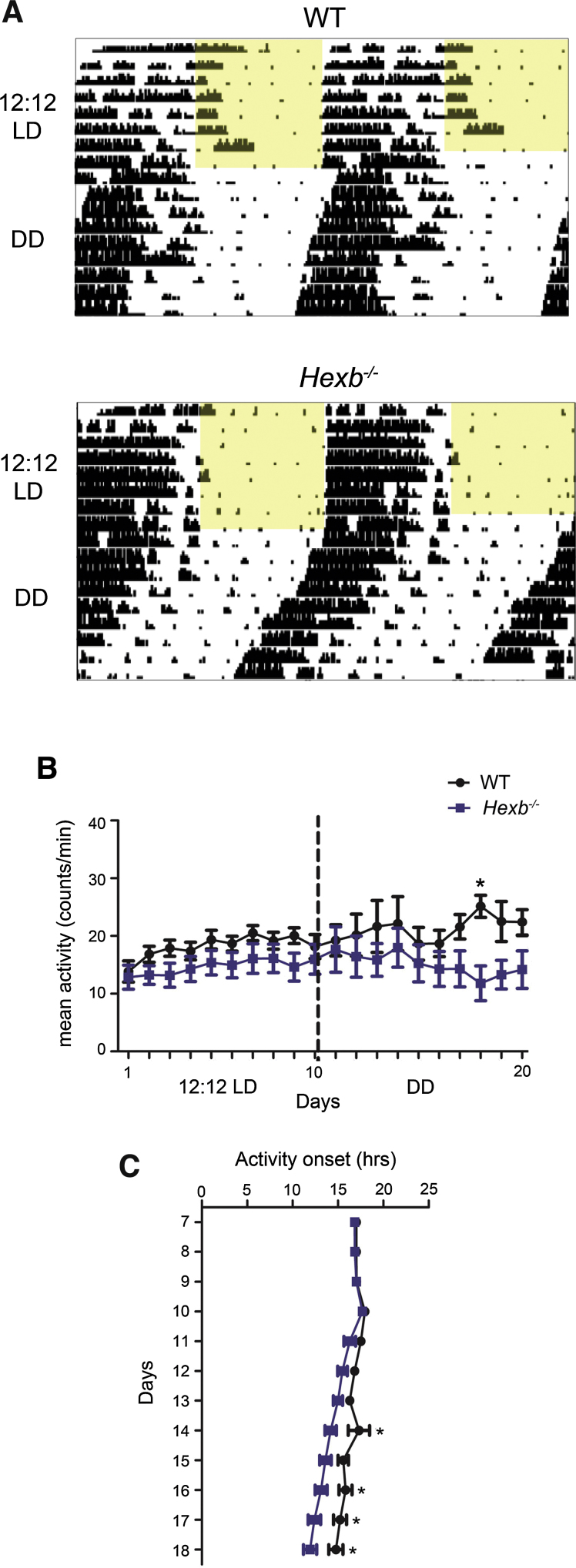
Wheel-running behaviour in *Hexb^−/−^* mutants in LD and DD. (A) *Hexb^−/−^* activity under a 12:12 LD cycle (from 7 weeks of age) followed by DD (*Hexb^−/−^* screen; Fig. S1 for details) versus WT controls. (B) *Hexb^−/−^* mutants show no difference in activity over each day in LD and at only one hour during DD (*n *= 6, Hexb screen 1) compared to WT controls (*n *= 7). (C) Free-running activity onsets under DD are significantly earlier in *Hexb^−/−^* mutants compared to WT. Data presented as mean ± SEM; *p < 0.05, ANOVA

Next we analysed *Hexb^−/−^* and WT littermate controls under the same circadian parameters of 12:12 LD and DD (Fig. S1). As observed in *Npc1^nih^* mice, *Hexb^−/−^* mutants displayed entrained 24-h rest/activity profiles, with no significant difference in the time of activity onset, circadian period, negative masking, or proportion of activity in the light phase compared to WT controls (Fig. 2A–C, Table 2, Fig. S3). *Hexb^−/−^* mutants displayed a slight reduction in activity in LD compared to controls, although this was only significant at one particular day in DD (Fig. 2B, Table 2). In contrast to *Npc1^nih^* mice, the alpha and average number of activity bouts per day in *Hexb^−/−^* mice was not significantly different to their WT controls (Fig. S2C–D). However, *Hexb^−/−^* mutants exhibited a small, but statistically significant shorter free-running period (mean tau = 23.3 +/− 0.05 h) versus WT littermates (mean tau = 23.7 +/− 0.06 h; *p* = 0.027) under DD (Fig. 2A and C); this shortened tau suggests that there may be deficits in core clock function in *Hexb^−/−^* mice.

**Fig. 3:**
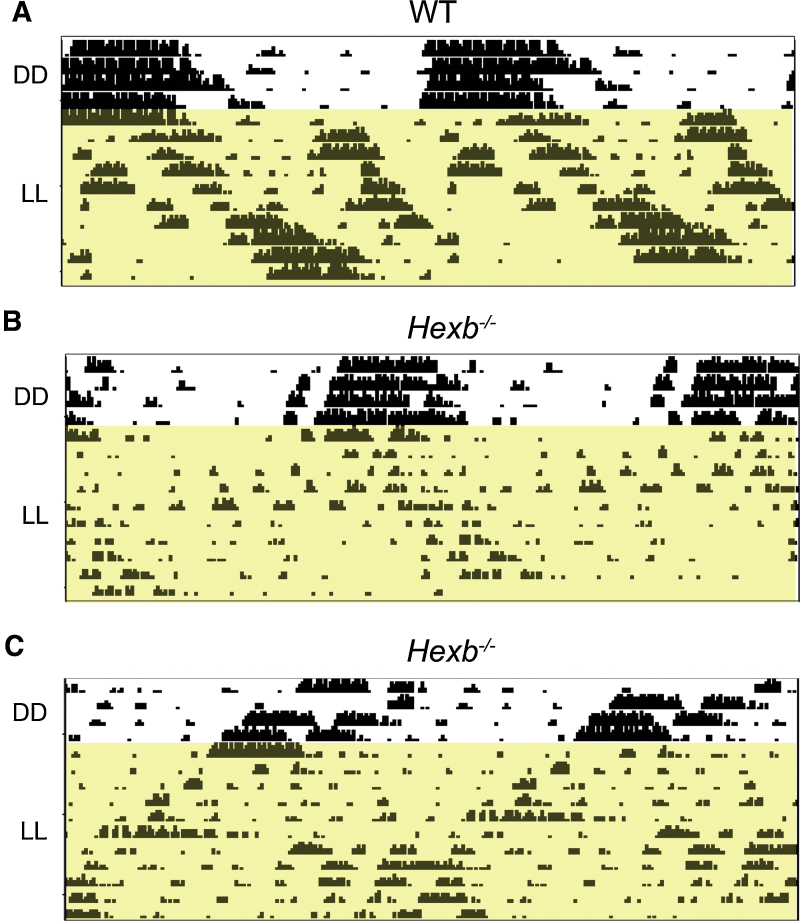
Wheel-running of *Hexb^−/−^* mutants under constant light (LL). After DD, *Hexb^−/−^* mutants and controls were place in LL (Fig. S1; *Hexb^−/−^* screen). (A and B) One third (2 of 6) of the *Hexb^−/−^* mutants displayed period lengthening as expeced in WT mice. (C) Two-thirds (4 out of 6) *Hexb^−/−^* mutants show a photoperiod of less than 24 h under LL.

*Hexb^−/−^* mutants display gait abnormalities at a later stage than *Npc1^nih^* mice, thus we were able to examine free-running behaviour under constant light (LL) in addition to DD in the same circadian screen (Fig. S1). LL is expected to cause a period lengthening effect as a measure of the circadian sensitivity to light and this paradigm was carried out to further investigate the activity onset differences observed in DD. Interestingly, we observed considerable variability in the wheel-running patterns of *Hexb^−/−^* mutants under LL (Fig. 3A–C). Despite progressive hypoactivity observed in all *Hexb^−/−^* animals, one third (2 of 6) showed period lengthening as seen in WT mice (Fig. 3A and B), whereas two-thirds of mutants showed a running pattern with a tau value continuing below 24-h, suggesting light-input pathways are disrupted in these animals towards end-stage disease (Fig. 3C).

**Fig. 4:**
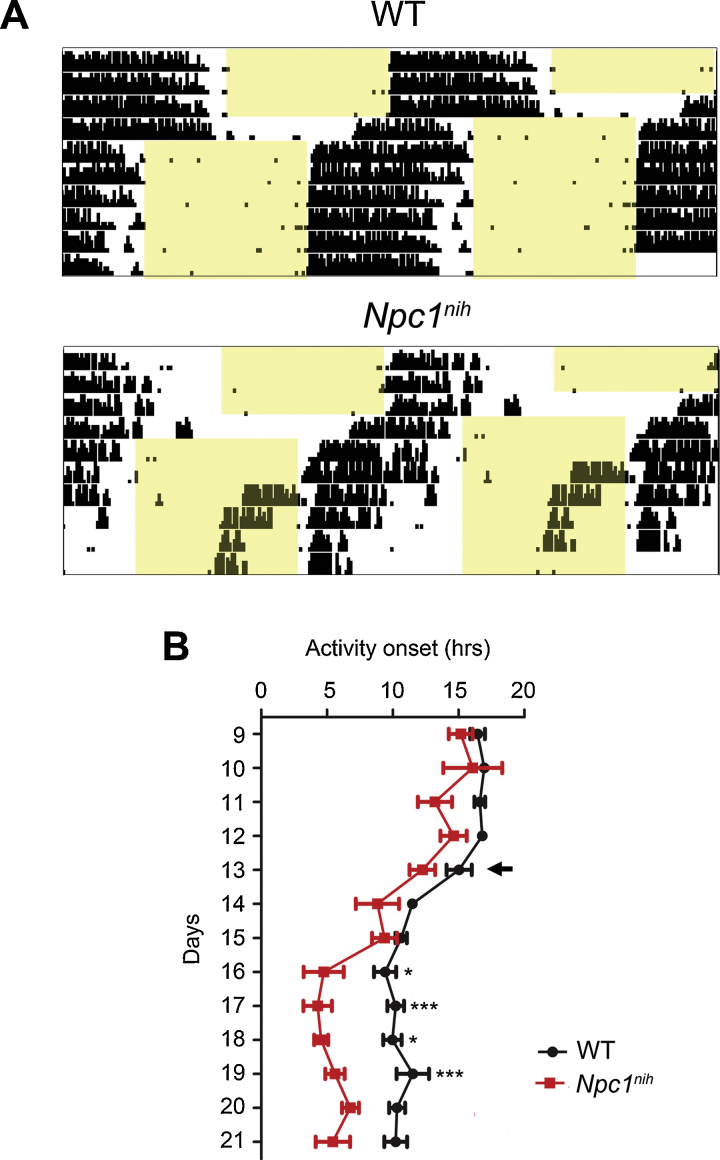
Phase-resetting behaviour in *Npc1^nih^* mutants. (A) *Npc1^nih^* activity onset following a six-hour phase advance was similar to WT for the first 2 days, however increasing onset variability was observed, indicating rhythm instability (*n *= 5). (B) Average activity onsets in *Npc1^nih^* mice were significantly different from WT 3 days after the phase advance (indicated with an arrow). Data presented as mean ± SEM; *p < 0.01, ***p < 0.0001, ANOVA.

Given the potentially fragmented activity in *Npc1^nih^* mice in DD, next we investigated the ability of the clock to entrain to a new LD cycle in these mutants using a jet-lag or phase-shift paradigm on second cohort of animals (screen 2, Fig. S1); after entrainment to 12:12 LD cycle, a 6-h phase advance (ZT0 = 0000 hr) lighting schedule was applied. *Npc1^nih^* mutants appeared to re-entrain to the new 12:12 LD cycle, however there was an large additional burst of activity approximately 5 h prior to lights-off (Fig. 4A). Consequently, there was a significant difference in the average activity onset time after 3 days of the new lighting schedule (Fig. 4B).

**Fig. 5:**
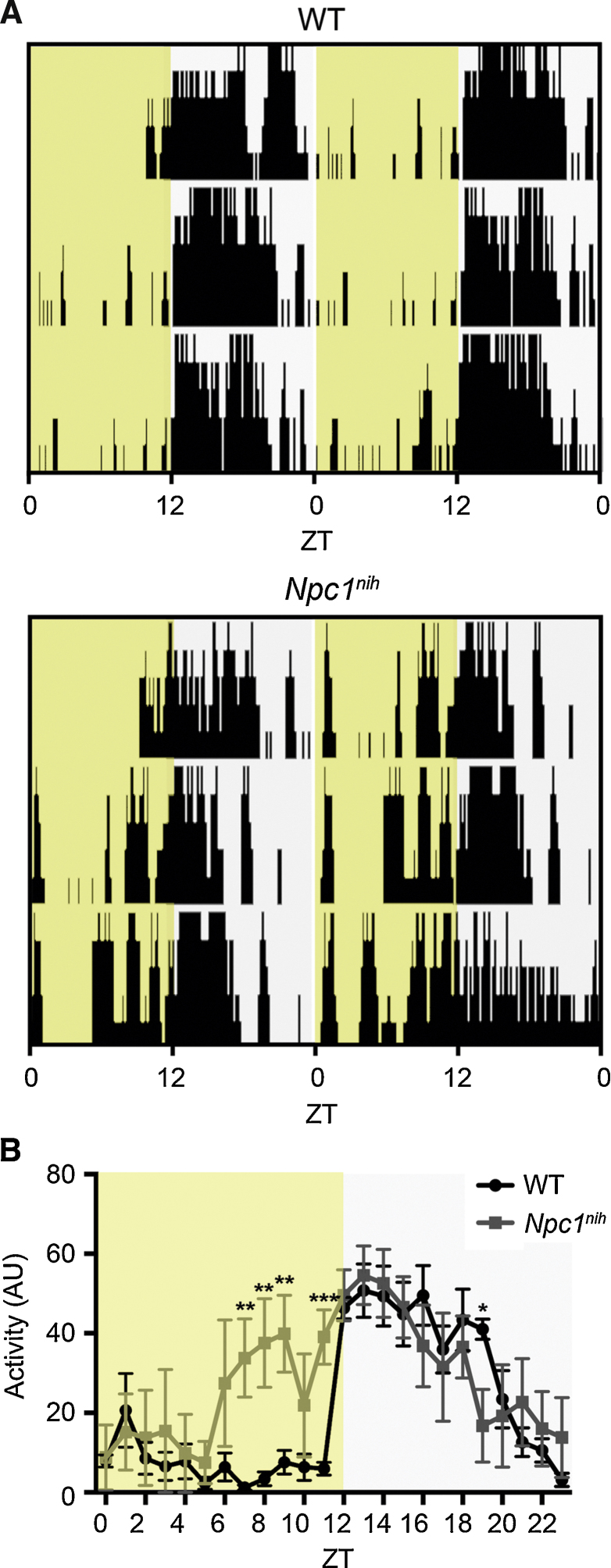
Passive-infrared (PIR) tracking in *Npc1^nih^* mutants in a 12:12 LD cycle. Each row represents a double-plot of the activity profile recorded over 24 h. (A) Activity bars are displayed in 10 min bins and yellow shading indicates light exposure. (B) Average activity over day 3 of the PIR screen. *Npc1^nih^* mutants show significant increase in light-phase activity compared to WT. **p < 0.01, ***p < 0.0001. (For interpretation of the references to colour in this figure legend, the reader is referred to the web version of this article.)

We speculated that the differences in activity levels and onset after the jet-lag protocol of *Npc1^nih^* mutants was due to their progressive, ataxic gait limiting their ability to run, or motivation to run, on the wheel. Therefore we carried out a daily activity screen using an third independent cohort of mice at 8.5 weeks of age using passive-infrared motion sensors [29]. Data were recorded over 5 days under a 12:12 LD cycle (ZT0 = 0600 hr) in standard homecages lacking a running wheel (Fig. 5A). This method was able to detect stable activity onsets in WT mice at ZT12 with limited activity in the light phase (Fig. 5A). Interestingly, after 2 days of acclimatisation to the cages, these data indicated a significant increase in activity of *Npc1^nih^* mice towards the second half of the light phase (ZT7-11) compared to WT controls (Fig. 5B); this is a similar pattern of activity to that seen in *Npc1^nih^* mutants under LD after the phase-advance paradigm (Fig. 4A). However, in the same 24-h period there was no significant reduction in average activity during the first 6 h of the dark phase in *Npc1^nih^* mice (ZT12-18) that would be indicative of a shortened alpha (Fig. 5B). Overall, these data from a short circadian screen using PIR sensors suggest that the reduced alpha observed initially under LD (Fig. S2A) could be due to the influence of the progressive ataxia on wheel-running capability.

### 3.2 Neuropathology

**Fig. 6:**
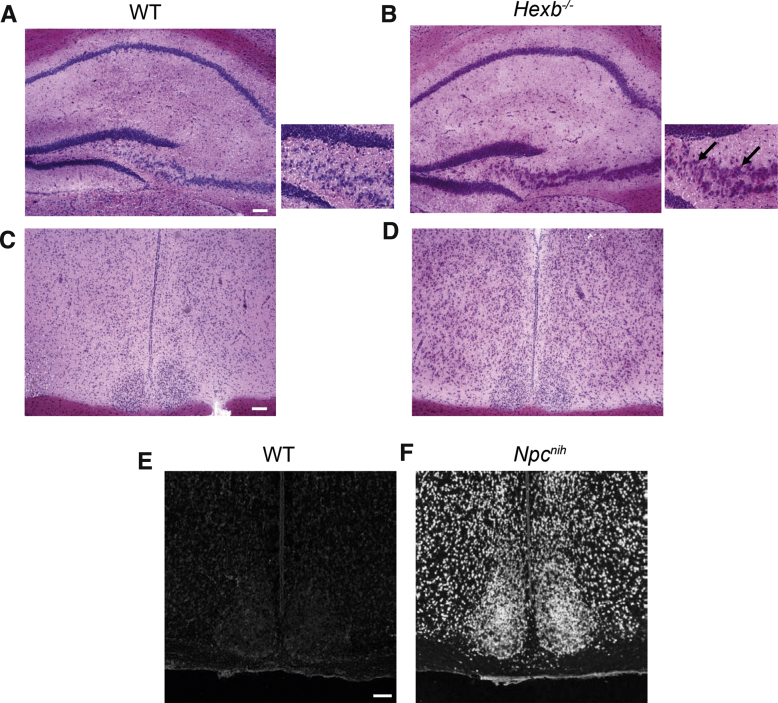
Neuropathology of LSD mouse models. (A–D) Representative light microscopy images of Periodic Acid–Schiff (PAS)-stained brain sections from *Hexb^−/−^* mutants and WT controls at 12 weeks of age. Stained sections are taken from the dentate gyrus (A, B) and the SCN (C, D). The glycosphingolipid (GSL) storage products stained with PAS appear pink. Arrows indicate PAS-positive neuronal inclusions in *Hexb^−/−^* mutants. (E, F) Representative fluorescent microscopy images of filipin-stained SCN sections from *Npc1^nih^* mutant and WT controls at 11 weeks of age. Scale bars: 100 μm.

Although previous studies have demonstrated widespread pathology in the brains of both *Npc1^nih^* and *Hexb^−/−^* mice [24], [33], we investigated whether the accumulation of cholesterol and glycosphingolipids occurs specifically in the SCN of *Npc1^nih^* and *Hexb^−/−^* mutants, respectively, as this may relate to the differences in wheel-running activity observed under constant conditions. First, PAS staining was used to determine the extent of lipid accumulation in *Hexb^−/−^* animals in comparison to WT littermates at 12 weeks of age, and strong staining was seen in the hippocampus as reported previously (Fig. 6A–B) [34]. Interestingly, there appeared to be a lack of lipid accumulation in the SCN (Fig. 6C–D). By contrast, in *Npc1^nih^* mice, extensive cholesterol accumulation was seen in all regions of the brain at 10 weeks of age using filipin staining, including the SCN itself (Fig. 6E–F). These data show that despite obvious pathology in the SCN, *Npc1^nih^* mutants are still able to maintain a normal circadian phenotype. Thus these data support the hypothesis that the reduced alpha and increased fragmentation observed in these mutants is likely to reflect early markers of disease pathology and ataxia rather than circadian defects.

### 3.3 Expression analysis

**Fig. 7:**
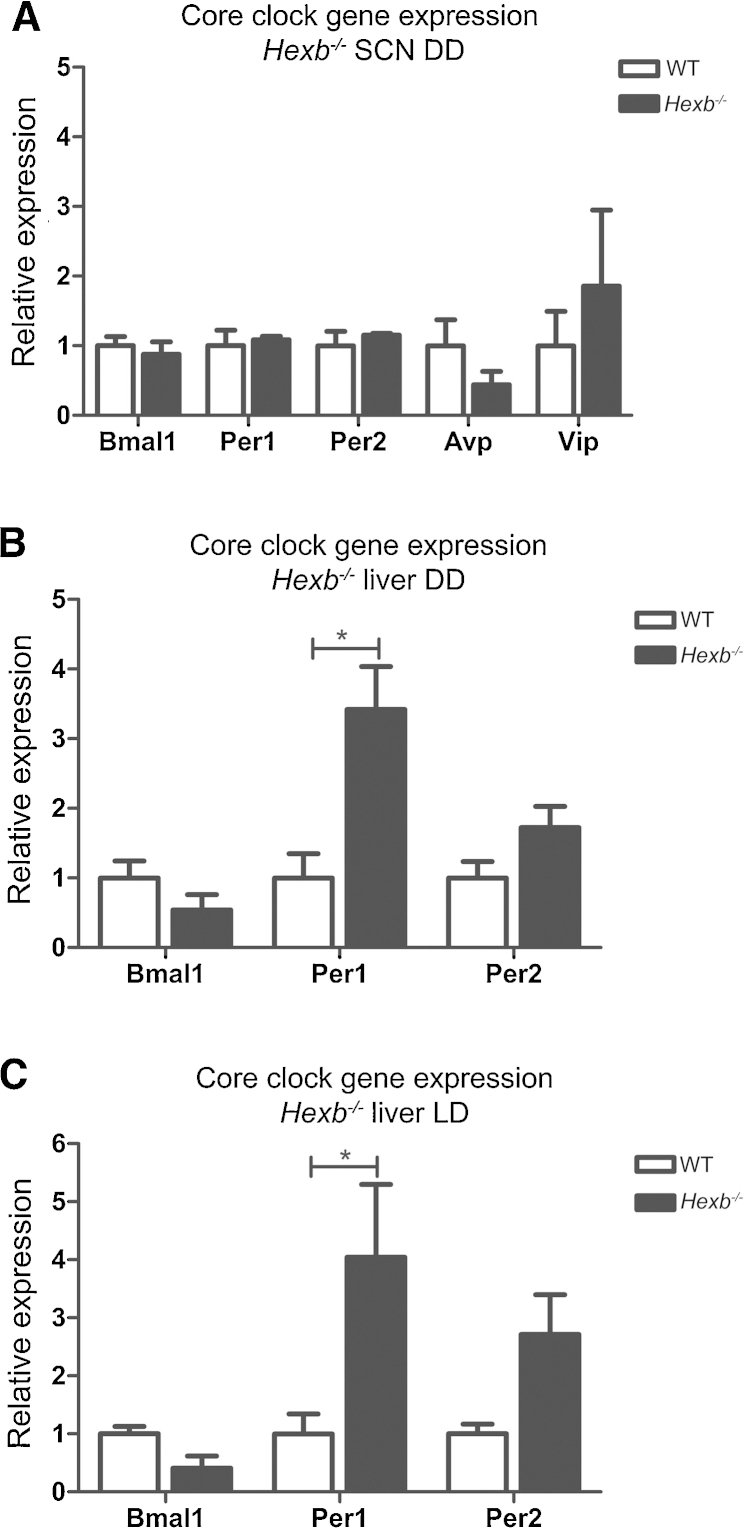
Clock gene expression in *Hexb^−/−^* mutants by RT-qPCR. (A) No significant difference in selected clock gene expression was observed in the SCN of *Hexb^−/−^* mutants versus WT at CT6 under DD (*n = 4–5*). *Hexb^−/−^* mutants show a significant differences in the expression of *Per1* in the liver at ZT6 under a 12:12 LD cycle (B) and at CT6 under DD compared (C) to WT (*n = 4–5*). Data are presented as mean ± SEM; *p = <0.001, ANOVA.

Due to alterations in the free-running activity of *Hexb^−/−^* mutants, we next investigated the core clock at the molecular level in these mice by measuring the expression of selected clock genes in both the SCN and liver at postnatal day (P) 72; a cohort of animals at 8 weeks of age were initially entrained to a 12:12 LD cycle (ZT0 = 0600 hr) for 14 days before being exposed to DD for 48 h prior to tissue collection at CT6 to assess expression changes during constant conditions where we observed potential behavioural changes. Quantitative RT-PCR from hypothalamic SCN tissue punches showed no significant changes in the relative expression levels of *Avp*, *Vip*, *Bmal1*, *Per1*, or *Per2* between *Hexb^−/−^* mutants and WT controls (Fig. 7A). Interestingly, a significant increase in the expression of *Per1* was observed in the liver of the same *Hexb^−/−^* mice compared to WT controls under DD (Fig. 7B). To determine whether these expression changes were restricted to constant lighting conditions, a separate cohort of 8-week old animals were subjected to a 12:12 LD cycle for 16 days and tissue as taken at ZT6. Here, a significant increase in *Per1* expression was again observed in the liver (Fig. 7C). These data suggest there is no global disruption of core clock gene expression in *Hexb^−/−^* mutants.

### 3.4 Retinal histopathology

**Fig. 8:**
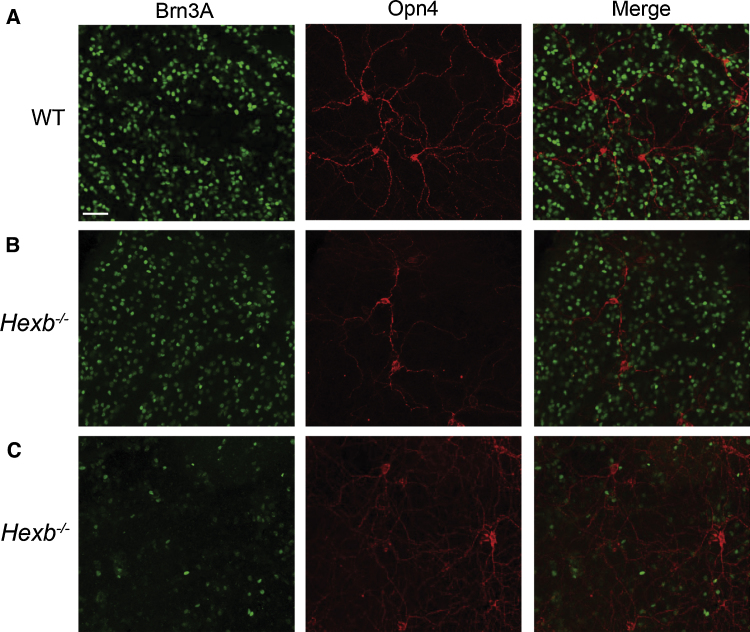
Retinal histopathology in *Hexb^−/−^* mutants. (A) Representative immunofluorescence images of Brn3a and melanopsin expression in WT ventral retina (*n *= 3). (B) Representative immunofluorescence images of Brn3a and melanopsin expression in *Hexb^−/−^* ventral retina (*n *= 3). (C) Some dorsal areas of *Hexb^−/−^* retina showed more significant disruption of Brn3a labelling. Scale bars: 50 μm.

The period lengthening effect of LL in *Hexb^−/−^* mutants suggests there is disruption to the light-input pathways in these animals. To investigate if the retinal ganglion cells (RGCs) mediating circadian responses to light are affected in this model, we assessed the melanopsin-expressing RGCs in *Hexb^−/−^* mutants. Immunofluorescence was used to compare the expression of the retinal ganglion cell marker Brn3a and the photosensitive retinal ganglion cell (pRGC) photopigment melanopsin in *Hexb^−/−^* mutants and WT littermate controls. Brn3a was widely expressed in RGCs located in the ganglion cell layer and displaced RGCs present in the inner nuclear layer, but absent from melanopsin expressing photosensitive RGCs (Fig. 8A and B). The anatomy and distribution of melanopsin staining was consistent with previous reports [31]. Multiple distinct subtypes of pRGCs were detected, and overall a significant dorsal ventral gradient in the distribution of pRGCs and levels of melanopsin expression was observed. Although large areas of *Hexb^−/−^* retina appeared grossly normal, some were found to contain areas of reduced and disrupted Brn3a staining consistent with RGC toxicity and the partial loss of these cells (Fig. 8C), with some showing more significant disruption of Brn3a labelling than others (Fig. S4A). This was typically evident as areas of cell clumping, abnormal cell morphology, and reduced levels of Brn3a expression. By comparison, levels of melanopsin expression observed in *Hexb^−/−^* retina was grossly similar to that observed in WT control retina (Fig. S4B). In areas of disrupted Brn3a labelling levels of melanopsin expression and the anatomy of pRGCs was typically normal, although some degree of pRGC disruption was observed in some of the most affected areas. Together, these data suggest that defects in retinal output does not entirely account for the variation in wheel-running towards end-stage in *Hexb^−/−^* mutants.

## 4 Discussion

Circadian phenotypes are reported increasingly in neurodegenerative disorders, including LSDs; however, the exact mechanisms linking circadian rhythm disruption and neuropathology remain largely unknown. Here we investigated the effect of mutations in the *Npc1* and *Hexb* genes on the circadian axis in mouse models of NPC1 and Sandhoff disease. Significant insights into the underlying biochemical/molecular basis of LSDs have been gained through the use of animal models [35]. NPC1 (*Npc1^nih^*) and Sandhoff (*Hexb^−/−^*) mutant mice recapitulate many features of the corresponding human disorders, including rapid onset of metabolic disease, excessive neuronal storage, and progressive motor and cognitive pathology [24], [26], [27]. Interestingly, despite some general phenotypic similarities between both mutants, we found some evidence for potential differences in their circadian phenotypes.

*Npc1^nih^* mutants showed normal entrainment to a 24-h LD cycle, and typical free-running circadian periods (tau) under constant dark for the background strain [36], suggesting no overt circadian disruption. However, *Npc1^nih^* mice did show some re-entrainment deficits following a six-h LD phase-advance at 9–11 weeks of age; this could be partially attributed to the extensive neuropathology and ataxia near end-stage in these mutants, causing fragmented wheel-running behaviour and apparent hypoactivity [33]. This was partially corroborated by the PIR tracking that also revealed an increase in activity in the light-phase of mutants at the same age under LD. Interestingly, we did not observe clear evidence for a reduction in total activity or shorter alpha in cages without running wheels in our PIR screen, supporting the notion that the progressive ataxic gait limits the wheel-running capability of *Npc1^nih^* mutants but not overall activity. A recent study has utilised the same PIR tracking system in parallel with video tracking and wheel-running assays in a mouse model of glutamate receptor dysfunction; these data also demonstrated that specific behavioural rhythms in mutant animals were dependent on the assay used to measure activity [37]. As such, when using running wheels to assay neurological mutants, the confounds of what is a voluntary motor task must be taken into account. Importantly, we were still able to detect robust free-running profiles close to end-stage disease in *Npc1^nih^* mutants as well as observing activity differences between genotypes from the first few days of screening, illustrating the ability of wheel-running assays to identify novel and subtle behavioural phenotypes [38].

Activity fragmentation was also evident in *Npc1^nih^* mice as shown by a small but significant increase in the average number of activity bouts per day, which may reflect an underlying defect that gives rise to sleep disturbances or simply represent the requirement to rest between bursts of activity due to ataxia. We also showed extensive cholesterol accumulation in the SCN, however this does not appear to influence the core molecular clock. Yet it has been hypothesised that neuropathology in the brainstem of *Npc1^nih^* mutants, such as decreased tyrosine-hydroxylase immunoreactivity in locus coeruleus, is indicative of an imbalance between cholinergic and monoaminergic activity, that may in-turn influence non-REM to REM sleep transitions [39]. Interestingly, previous studies report fragmented and disorganised sleep in NPC1 patients [20], [21], although further experiments using telemetry or longer-term video tracking would be required to analyse sleep in LSD models and indentify parallels with patients.

We showed that *Hexb^−/−^* mutants display some evidence for aberrant circadian behaviours, despite an apparent lack of glycosphingolipid accumulation (PAS staining) in the SCN; under the DD paradigm a small but significant reduction in tau was observed, indicative of endogenous clock dysfunction. No differences were seen in the expression of selected core clock genes in the SCN under DD conditions, however. In *Hexb^−/−^* mice, the pathology is widespread and not simply focussed in the cerebellum, with the cells in the cerebral cortex, thalamus, striatum, hippocampus also showing accumulation of GM2 ganglioside in addition to neuroinflammation and widespread neuronal death [24], [34]. Therefore, although it is unclear how the function of these specific brain regions will be affected, it is possible that the widespread nature of the GM2 pathology may lead to circadian feedback to the clock. The differences in *Per1* expression in the *Hexb^−/−^* liver in both DD and LD could be an indirect result of liver pathology influencing immediate early genes in mutant mice, yet this may also demonstrate uncoupling of peripheral clocks. As clock gene expression appeared normal in the hypothalamus of *Hexb^−/−^* mice it unlikely that this could, in turn, feedback to the SCN and influence free-running behaviour. It is becoming increasingly evident, however, that close interaction between central and peripheral clocks is necessary to maintain robust circadian rhythms of physiology and metabolism [40], [41].

A range of defective autophagic lysosomal pathways occur in LSDs; from the abnormal enzymatic processing in Sandhoff disease to the non-enzymatic deficiencies as seen in NPC1 [2], [42]. The specific pathways affected result in differential patterns of neurodegeneration, suggesting that specific neuronal subtypes likely contribute to the subtle circadian behavioural changes observed in *Npc1^nih^* and *Hexb^−/−^* mice. Previous behavioural studies of other LSD mouse models have also shown contrasting circadian profiles. In a model of mucopolysaccharidosis (MPS) III, mice are deficient for the lysosomal enzyme *N*-acetyl-glucosaminidase, and lysosomal storage is observed in multiple brain regions including the SCN [43]. These mutants demonstrate a significant increase in activity during the light phase in comparison to controls under a LD cycle, but no free-running deficits [44]. By constrast, mice lacking lysosomal α-mannosidase, a model of the LSD α-mannosidosis were reported to have no circadian abnormalities [45]. Thus it is noteworthy that *Npc1*, *Hexb* and many other genes implicated in LSDs have a fluctuating daily expression profiles at the RNA level [46], suggesting that the biological clock itself may impart additional regulation upon critical lysosomal enzymes and metabolites that will ultimately influence circadian behaviour.

The SCN is innervated by intrinsically photosensitive retinal ganglion cells mediating non-image forming responses to light [47]. Our data show these melanopsin expressing cells showed signs of stress and damage in some areas of the retina in *Hexb^−/−^* mice. Immunostaining from WT mice showed a pattern of Brn3 and melanopsin staining consistent with previous reports from WT retina [31]. A significant reduction in Brn3a expressing RGCs was observed in the retina of *Hexb^−/−^* mice in comparison to WT mice. However, these RGCs do not project directly on to the SCN, rather form part of the principal thalamocollicular visual pathway mediating cortical vision [48]. This is in support of previous studies which have illustrated extensive GM2 deposition in RGCs, vacuolation in retinal neurons, and disruption of the retinal pigment epithelium in *Hexb^−/−^* mice [49], [50], [51]. Importantly, these animal studies also correlate with the vision loss identified in Sandhoff disease patients [52]. Previous work has shown that even in the absence of light the eyes still exert effects on circadian behaviour, and key markers of SCN function are unaffected following enucleation [53]. The retinal pathology observed in the *Hexb^−/−^* mutants may explain the small, but significant, reduction of tau in DD, and explain the lack of core clock gene disruption in the SCN associated with this. Our *Hexb^−/−^* data suggests mild hypoactivity coupled with end-stage retinal ganglion cell disruption in these mutants. Previous reports suggest the effects of the retinal circadian system on the free-running period is difficult to predict, and it is unknown whether changes observed are a result of the interaction between the retinal and circadian system or due to photoreceptor degeneration [54].

## 5 Conclusion

The molecular circadian feedback loop regulates the expression of clock-controlled genes in a rhythmic manner, resulting in the oscillation of tissue-specific metabolic and physiological functions [55]. Here we describe two mutant mouse models of LSD that display some minor differences in their circadian behaviour and potential perturbations in the peripheral molecular clock in *Hexb^−/−^* mutants. Despite the extensive CNS pathology observed in some LSDs, the hypothalamic oscillatory network may remain intact and exhibit only minor defects in these disorders. As we have demonstrated, specific populations of neurons are differentially affected depending on the nature of the disease [56], indicating the alternative mechanisms that underlie the neuropathology in LSDs may influence the rest/activity profiles observed in this group of disorders. More research will be required to understand this relationship and ascertain if the timing of lysosomal enzyme activity could influence therapeutic approaches [46].

## Figures and Tables

**Table 1:** Circadian activity parameters for *Npc1^nih^* mice during LD and LL.

Parameter	WT mean (±SEM)	*Npc1^nih^* mean (±SEM)	Significance
Activity onset (hours)	16.1 (0.23)	15.6 (0.38)	NS
Light activity (% of total activity)	12.34 (2.167)	16.59 (3.196)	NS
Circadian period (hours)	24.05 (0.05)	24.04 (0.07)	NS
Total activity counts/day	31083 (2033)	14178 (1051)	*p* < 0.0001

**Table 2:** Circadian activity parameters for *Hexb^−/−^* mice during LD.

Parameter	WT mean (±SEM)	*Hexb^−/−^* mean (±SEM)	Significance
Activity onset (hours)	17.10 (0.04)	16.92 (0.05)	NS
Light activity (% of total activity)	8.185 (3.40)	3.319 (1.12)	NS
Circadian period (hours)	23.84 (0.04)	23.95 (0.02)	NS
Total activity counts/day	25767 (1312)	20847 (2939)	NS
