# Supplementary material for: Circadian profiling in two mouse models of lysosomal storage disorders; Niemann Pick type-C and Sandhoff disease
Source: Behav Brain Res. 2016 Jan 15;297:213–23. doi: 10.1016/j.bbr.2015.10.021 (PMC4678117; doi:10.1016/j.bbr.2015.10.021)
Supplement: Supplementary file 1 [file mmc1.doc]

| **Primer** | **5’-primer (5’-3’)** | **3’-primer (5’-3’)** | **Annealing temp (°C)** | **Product length (bp)** |
| --- | --- | --- | --- | --- |
| *Gapdh:* | TGTGTCCGTCGTGGATCTGA | CCTGCTTCACCACCTTCTTG | 68 | 77 |
| *Per1* | GGGTGAGATTCGTCATTGAACTTG | AGGACATTGGCACACTGGAAAGAG | 68 | 119 |
| *Bmal1* | GAGGTGCCACCAACCCATAC | AGTCAAACAAGCTCTGGCCAA | 67 | 207 |
| *Per2* | CCAGGATGTGGGTGTCTTCT | GAGACCTGAACCTGCAGAGG | 64.0 | 85 |
| *Avp* | CAGGATGCTCAACACTACGC | CTCTTGGGCAGTTCTGGAAG | 68 | 82 |
| *Vip* | TTTCACCAGCGATTACAGCAG | GCTGATTCGTTTGCCAATGAG | 68 | 86 |

**Table S1:** Primers used for qRT-PCR
