# Supplementary material for: Circadian profiling in two mouse models of lysosomal storage disorders; Niemann Pick type-C and Sandhoff disease
Source: Behav Brain Res. 2016 Jan 15;297:213–23. doi: 10.1016/j.bbr.2015.10.021 (PMC4678117; doi:10.1016/j.bbr.2015.10.021)
Supplement: Supplementary file 2 [file mmc2.doc]

**Supplementary Figure legends:**


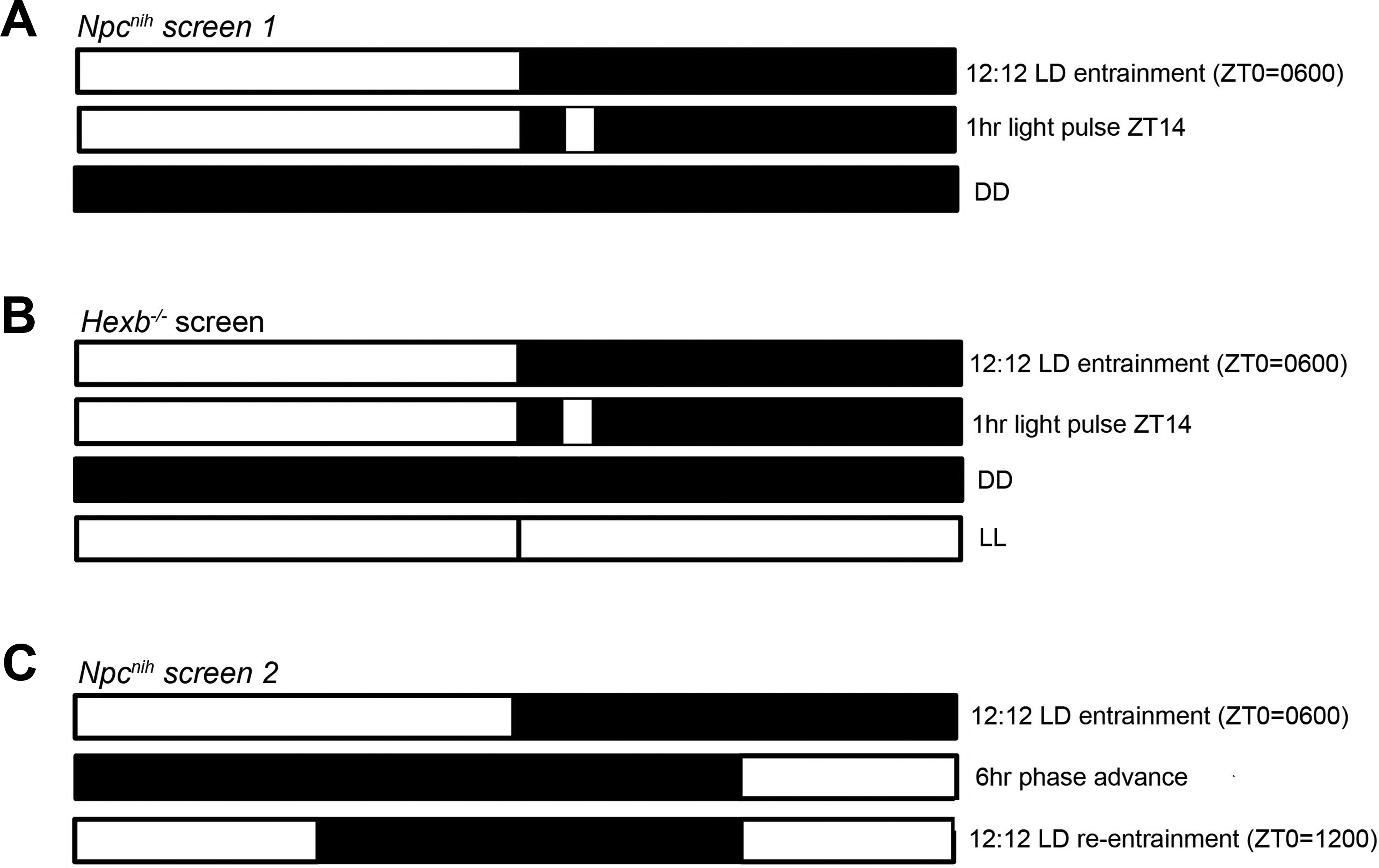


Fig. S1: Circadian wheel-running screens used in this study. (A) *Npc1^nih^* circadian screen 1. (B) *Hexb^-/-^* circadian screen. (C) *Npc1^nih^* circadian screen 2. The Zeitgeber time (ZT) of all environmental lighting changes is indicated. DD: constant dark, LL: constant light. LD: light/dark.


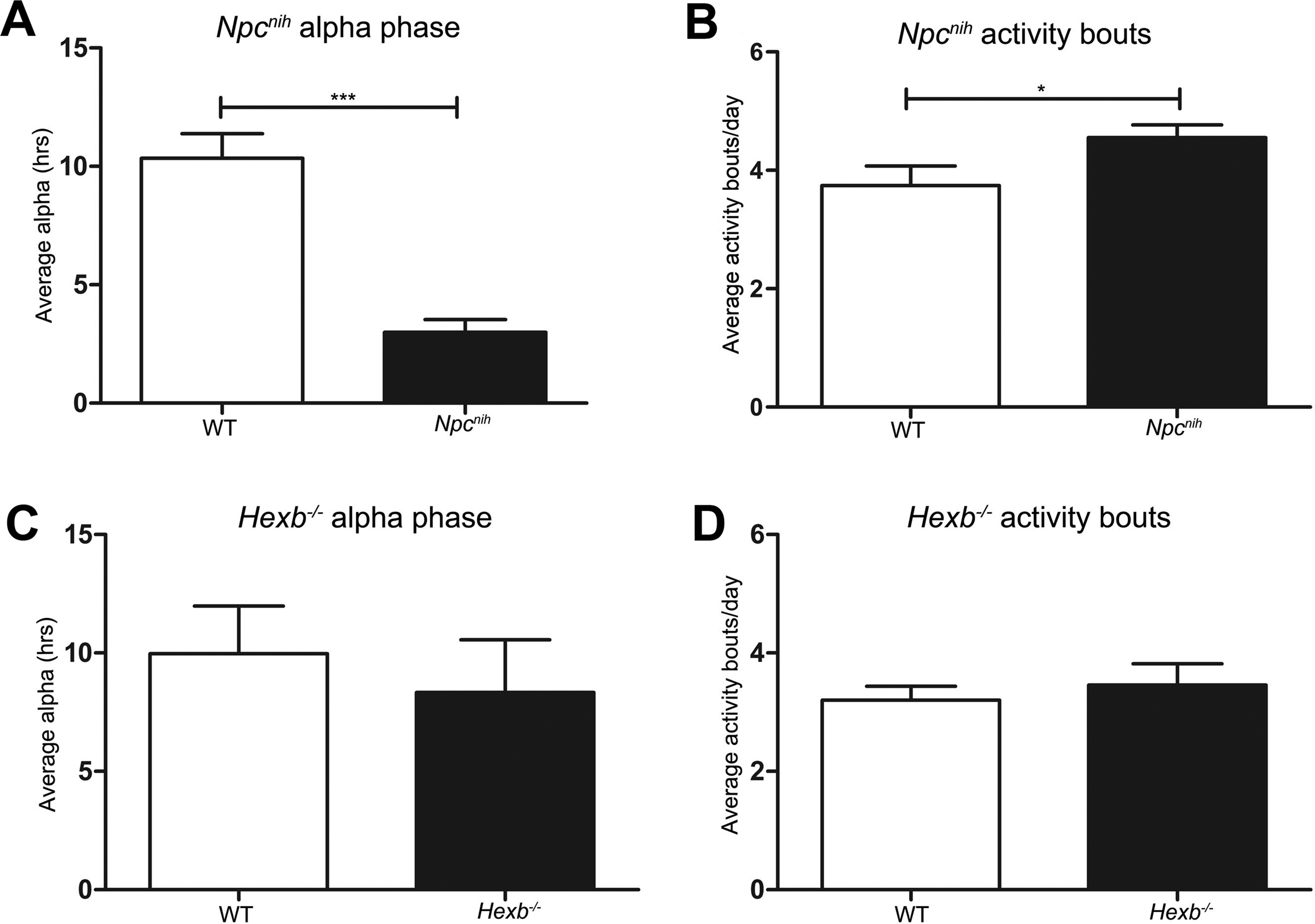


Fig. S2: Wheel-running activity measurements. (A) *Npc1^nih^* mice demonstrated a shorter active phase (alpha) compared to WT mice, accompanied by an increase in the number of activity bouts per day during LD (B) (*n*=11). (C, D) No significant differences were observed in the length of the active phase or the number of bouts in *Hexb^-/-^* mice versus controls (*n*=7). Data are presented as mean ± SEM; **p*<0.05, ****p*<0.001, ANOVA.


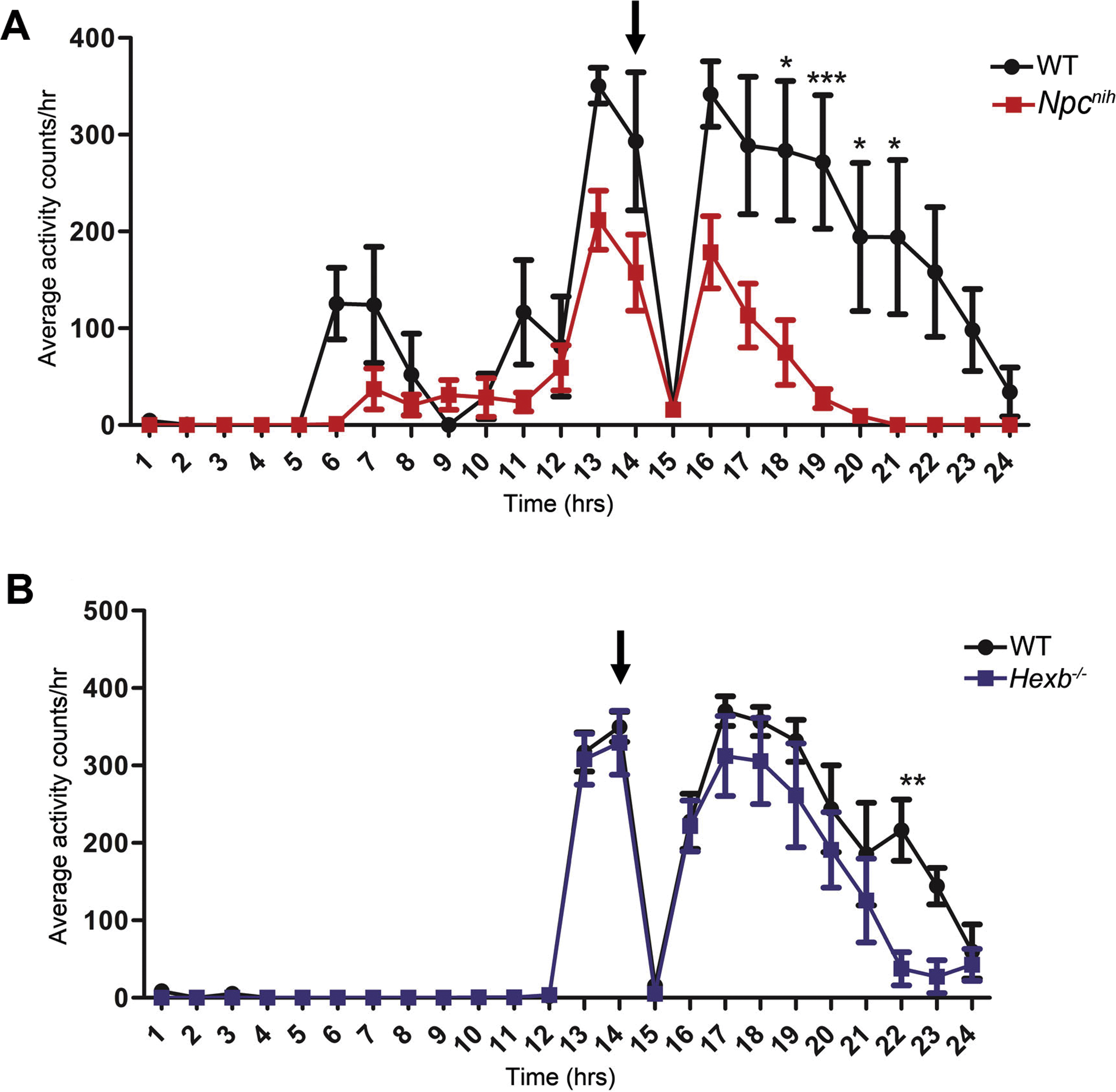


Fig. S3: Negative masking. Average activity counts are shown under 12:12 LD over a 24-hour period with lights off at ZT12. Both *Npc^nih^* (A) and *Hexb^-/-^* mice (B) at 8-9 weeks of age displayed a normal suppression of activity in response to a 1 hour light pulse given at ZT14 (arrow). Hypoactivity in the dark phase is also observed in both mutants. Data presented as mean ± SEM; **p*<0.05, ***p*<0.01, ****p*<0.0001, ANOVA.


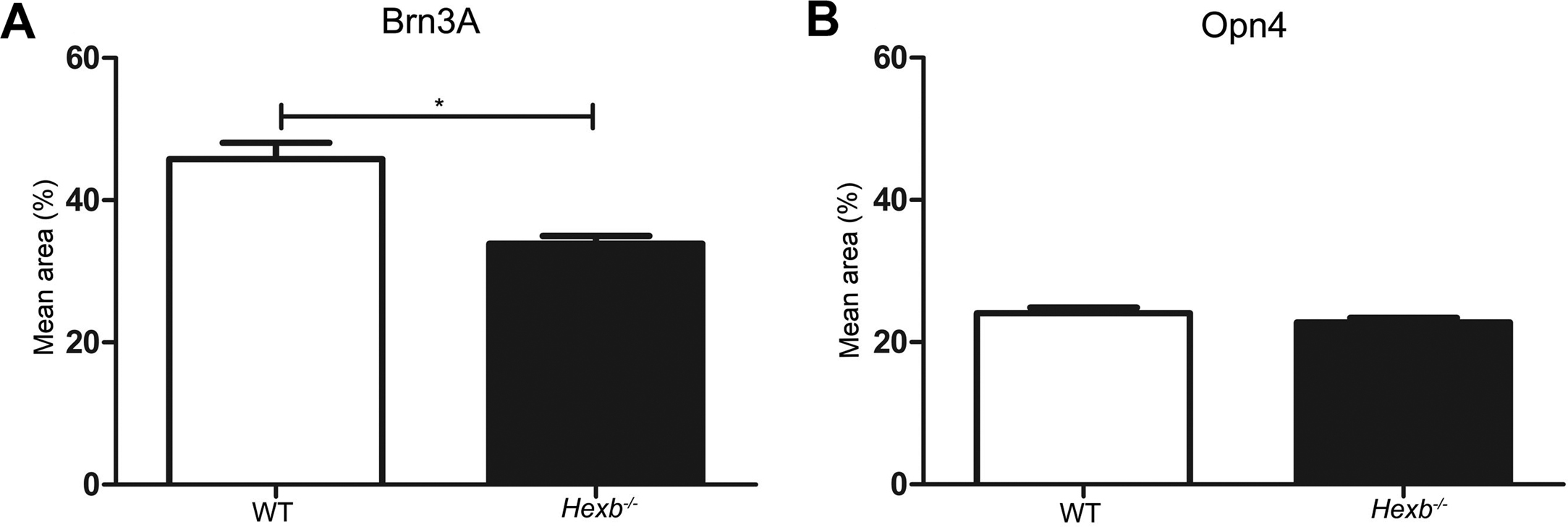


Fig. S4: Retinal histopathology. (A) Quantification of Brn3a expression in WT and *Hexb^-/-^* retina (*n*=3). (B) Quantification of Opn4 expression in WT and *Hexb^-/-^* retina (*n*=3). Data values represent the percentage area of each confocal image showing positive immunostaining above background. Values were calculated by performing automated threshold analysis followed by measurement of area above threshold using standard setting in Image J (NIH). Analysis performed on *n*=26 images (*Hexb^-/-^*) and *n*=14 images (WT) collected from *n*=3 retina for each group. Data presented as mean ± SEM; ****p*<0.001, Student’s t-test.
